# Financial toxicity in cancer patients treated with radiotherapy in Germany—a cross-sectional study

**DOI:** 10.1007/s00066-022-01936-z

**Published:** 2022-04-25

**Authors:** Alexander Fabian, Justus Domschikowski, Wolfgang Greiner, Gunnar Bockelmann, Elias Karsten, Alexander Rühle, Nils H. Nicolay, Anca L. Grosu, Jürgen Dunst, David Krug

**Affiliations:** 1 Department of Radiation Oncology, University Hospital Schleswig-Holstein, Campus Kiel, Arnold-Heller-Str. 3, 24105 Kiel, Germany; 2 Department of Radiation Oncology, University Hospital Freiburg, Robert-Koch-Str. 3, 79106 Freiburg, Germany; 3 Hochschule Wismar, University of Applied Sciences—Technology, Business and Design, Philipp-Müller-Str. 14, 23966 Wismar, Germany; 4 Department of Radiation Oncology, West Coast Hospital, Esmarch-Str. 50, 25746 Heide, Germany

**Keywords:** Financial distress, Oncology, Patient preference, Quality of life, Supportive care

## Abstract

**Purpose:**

Financial toxicity arises in cancer patients from subjective financial distress due to objective financial burden from the disease or treatment. Financial toxicity associates with worse outcomes. It has not been described in cancer patients undergoing radiotherapy in Germany and its publicly funded health system. In this context, we therefore investigated the prevalence of financial toxicity, associated risk factors, and patient preferences on communication of financial burden.

**Methods:**

We conducted a preregistered (10.17605/OSF.IO/KH6VX) cross-sectional study surveying patients at the end of their course of radiotherapy in two institutions. Objective financial burden was assessed by direct costs and loss of income. Financial toxicity was measured by subjective financial distress per EORTC QLQ-C30. We used Spearman’s correlation and Fisher’s exact test for univariate analysis, an ordinal regression for multivariate analysis. A *p*-value < 0.05 was considered statistically significant.

**Results:**

Of the 100 patients participating in the study, 68% reported direct costs, 25% loss of income, and 31% subjective financial distress. Per univariate analysis, higher subjective financial distress was significantly associated with active employment, lower quality of life, lower household income, higher direct costs, and higher loss of income. The latter three factors remained statistically significant in the multivariate analysis. A relative majority of the patients welcomed communication regarding financial burden with their radiation oncologist.

**Conclusion:**

Financial toxicity is prevalent in cancer patients treated with radiotherapy in Germany. The reported risk factors may help to identify patients at risk. Future studies should validate these results and investigate interventions for financial toxicity to potentially improve outcomes.

**Supplementary Information:**

The online version of this article (10.1007/s00066-022-01936-z) contains supplementary information, which is available to authorized users.

## Introduction

Financial toxicity in cancer patients is an emerging side effect of the disease itself and/or its treatment. Although different concepts and definitions exist, a common one defines financial toxicity as a potential consequence of subjective financial distress arising from cancer-related objective financial burden [1]. Objective financial burden, in turn, may be due to direct costs or indirect costs such as loss of income. Financial toxicity has been associated with suboptimal patient satisfaction and impaired treatment outcomes such as overall survival [2, 3]. Therefore, there is a strong rationale to detect and prevent or alleviate financial toxicity in cancer patients.

Cancer patients may be exposed to financial toxicity differently depending on the country they receive care in, as health care systems vary. Financial toxicity has been charted early and extensively in the US [4, 5]. In fact, the pertinent term of financial toxicity was introduced by Zafar and colleagues describing “the negative personal financial impact of cancer care” on the patient level in a US pilot study [6]. In analogy to physical toxicity of cancer care, they proposed that certain patients might be more prone to financial toxicity than others. Already this pilot study, however, found financial toxicity not only in uninsured but also in insured US cancer patients. Accordingly, evidence continues to grow that cancer patients face financial toxicity also in publicly funded health care systems [7]. An Italian study, for example, reported financial toxicity in 22.5% of 2735 cancer patients treated in different prospective clinical trials [3]. In this study, financial toxicity was significantly associated with an increased risk of death (hazard ratio [HR] 1.2, 95% confidence interval [CI] 1.05–1.37, *p* = 0.007). Furthermore, a French study described some degree of subjective financial distress in 51% of 143 patients with advanced cancer in a cross-sectional study [8]. Data on financial toxicity of cancer patients in Germany, however, is scarce. A narrative review on existing German studies described that loss of income is more influential compared to direct costs concerning objective financial burden [9]. Furthermore, they summarize risk factors for subjective financial distress including low household income, reduced employment, very young age at cancer diagnosis, or recurrent disease. Yet the authors conclude that additional studies are needed in Germany to evaluate the extent, risk factors, and consequences of financial toxicity.

In addition, there is a paucity of studies on financial toxicity in cancer patients treated with radiotherapy in general and, to our knowledge, there is no study from Germany [10, 11]. Yet at least 50% of all cancer patients in Europe receive radiotherapy during the course of their disease [12]. These patients may experience financial issues specific to radiotherapy such as costs for transportation and supportive care or loss of income due to extended duration of their treatment.

Therefore, we conducted a preregistered cross-sectional study on financial toxicity in cancer patients treated with radiotherapy in Germany. In this context, our aims were first to assess the prevalence of financial toxicity, second to identify factors associated with financial toxicity, and third to investigate the patient knowledge and preferences on communication of financial burden.

## Materials and methods

### Study design

We conducted an exploratory, survey-based, cross-sectional study in two radiation oncology departments in Germany. One department is part of a tertiary university hospital (University Hospital Kiel). The other department is affiliated and located at a peripheral teaching hospital (West Coast Hospital Heide). The study was performed during 60 consecutive days in the former and 30 consecutive days in the latter center in August and September 2021. The study protocol as well as the questionnaire were preregistered on The Open Science Framework (10.17605/OSF.IO/KH6VX) [13].

### Patient eligibility

Patients were eligible to participate if they (i) were about to complete a course of radiotherapy for cancer (last radiotherapy fraction ±2 days), (ii) could fill out a questionnaire, (iii) were > 18 years, (iv) had not participated in the study for another course of radiotherapy, and (v) gave informed consent. All potentially eligible patients were invited to participate during the period mentioned above. To address participation bias, the number of eligible patients who refused study participation was recorded.

### Consent to participate

All participants included in the study gave informed consent prior to enrolment.

### Questionnaire, variables, and outcomes

The questionnaire was designed for the purposes of the study and pilot-tested on three voluntary, potentially eligible patients. As the survey was anonymous, all study data were derived from the patient-reported survey except for the variable “treatment intent” which was documented by the treating radiographers who received the sealed questionnaire from the patient and who were not involved in data analysis. The survey included questions on patient characteristics as covariables as well as questions on outcome parameters (Supplementary Information 1). Patient characteristics included sociodemographic data, clinical data, and the global health status/quality of life per EORTC QLQ-C30 questionnaire (questions 29 + 30) [14]. Global health status/quality of life was scored according to the EORTC QLQ-C30 scoring manual [15]. To the best of our knowledge, there is no tool in German to assess financial toxicity by means of a single effect size. Therefore, we analyzed objective financial burden and subjective financial distress separately following the conceptual framework of financial toxicity by Witte and colleagues [1]. Outcome parameters for objective financial burden were assessed by a question on additional costs (direct costs) as well as loss of income (indirect costs) in the context of radiotherapy. The outcome parameter for subjective financial distress was a validated question on financial difficulties per EORTC QLQ-C30 (question 28) [14]. Additional outcome parameters included 5‑point Likert-scaled questions on the degree of feeling well-informed and the patient preference on communication regarding financial burden in the context of radiotherapy. These two questions were not formally validated but based on a previous, similar cross-sectional study [16].

### Statistical analysis

For statistical analysis, descriptive statistics were used to characterize the study population. Univariate analysis of associations between covariables and outcome parameters were performed by Spearman’s correlation for ordinally scaled and Fisher’s exact test for nominally scaled covariables. Fisher’s exact test was chosen because of expected cell counts lower than five in the respective contingency tables. Missing data were excluded in a pairwise manner. We did not adjust for multiple testing due to the exploratory nature of our study [17]. Ordinal regression analysis was used to assess a multivariate model of independent covariables and an ordinally scaled outcome parameter as dependent variable. A *p*-value below 0.05 was considered statistically significant. The software JASP v0.16 (JASP Team [2022], Amsterdam, The Netherlands) and IBM SPSS Statistics v27.0 (IBM Corp. [2020], Armonk, NY, USA) were used for statistical analyses.

## Results

### Patient participation and characteristics

During the study period, 213 cancer patients completed a course of radiotherapy. Of these, 187 were potentially eligible, and 100 patients completed the questionnaire, resulting in a participation rate of 53.5%. The completion rate for all variables of the questionnaire was 96%. Patient characteristics are shown in Table 1.

**Table 1:** Patient characteristics (*n* = 100)

Characteristics		% (*n*)
Total number of patients		100% (100)
Sex	Male: female	57:43
Age		Median: 65; IQR: 15
Marital status	Lives alone	26% (26)
	Lives with partner	73% (73)
Number of persons living in household	1	22% (22)
	2	62% (62)
	3	11% (11)
	> 3	6% (6)
Education	< 10 years of school	25% (25)
	10 years of school	39% (39)
	> 10 years of school	34% (34)
Health insurance	Public health insurance	77% (77)
	Of these, exempt from copayments	13% (10)
	Private health insurance	22% (22)
Employment status	Employed/self-employed	33% (33)
	Unemployed	11% (11)
	Retired	54% (54)
Net household income per month	< 1300 €	15% (15)
	1301–1700 €	15% (15)
	1701–2600 €	27% (27)
	2601–3600 €	20% (20)
	3601–5000 €	8% (8)
	> 5000 €	8% (8)
Tumor entity	Breast cancer	24% (24)
	Prostate cancer	18% (18)
	Brain tumor (primary or secondary)	10% (10)
	Head and neck cancer	8% (8)
	Lung cancer	8% (8)
	Rectal cancer	8% (8)
	Esophageal cancer	5% (5)
	Other	16% (16)
Treatment intent	Curative	57% (57)
	Palliative	43% (43)
Concomitant chemotherapy	Yes	25% (25)
	No	75% (75)
Hospitalized during radiotherapy	Yes (in part or throughout)	22% (22)
	No	76% (76)
Global health status/QoL	Per EORTC QLQ-C30	Mean: 52; SD: 22.6

Proportions are indicated as percentages. Absolute numbers are given in parentheses. Numbers may not add up to 100% due to rounding error or missing values. Concerning publicly insured patients, copayments are additional out-of-pocket costs for otherwise reimbursed services or products such as hospital stays or drugs. Patients with low income may apply for an exemption from copayments

*IQR* interquartile range; *QoL* quality of life; *SD* standard deviation

### Prevalence of objective financial burden and subjective financial distress

**Fig. 1:**
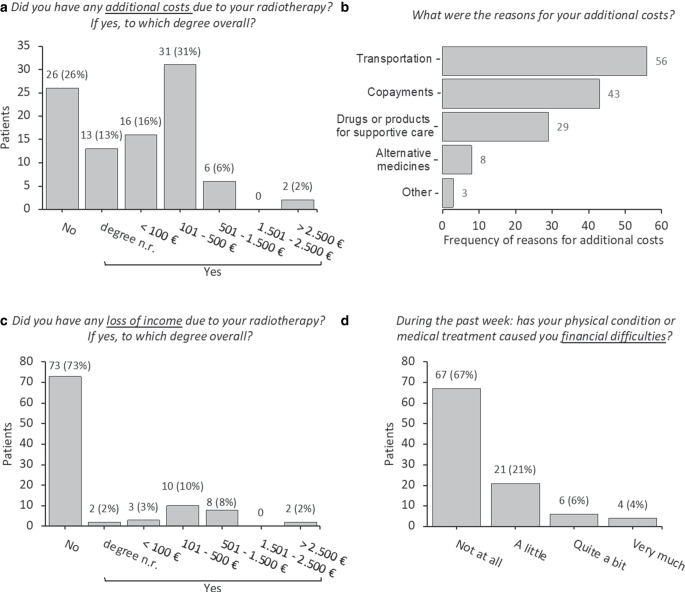
Prevalence of objective financial burden (**a**–**c**) and subjective financial distress per question 28 of the EORTC QLQ C-30 (**d**) in 100 patients treated with radiotherapy. Numbers may not add up to 100 (100%) due to missing values. Concerning publicly insured patients, copayments (in **b**) are additional out-of-pocket costs for otherwise reimbursed services or products such as hospital stays or drugs. The category “drugs or products for supportive care” (in **b**) involves only nonrefunded drugs or products for supportive care of radiotherapy to be paid entirely by the patient. *n.r.* not reported

We investigated the prevalence of objective and subjective financial distress separately. Objective financial burden as additional costs (direct costs) was denied by 26 (26%), affirmed by 68 (68%) and left unanswered by 6 (6%) patients (Fig. 1a). Costs for transportation, copayments, and nonrefundable drugs or products for supportive care were most prevalent (Fig. 1b). Objective financial burden as loss of income (indirect costs) was denied by 73 (73%), affirmed by 25 (25%), and left unanswered by 2 (2%) patients (Fig. 1c). Subjective financial distress as per question 28 of the EORTC QLQ-C30 questionnaire was not felt by 67 (67%), indicated by 31 (31%), and left unanswered by 2 (2%) patients (Fig. 1d).

### Factors associated with subjective financial distress

Next, we analyzed factors associated with financial toxicity. We chose the degree of subjective financial distress as proxy for financial toxicity, as subjective financial distress is the outcome closest to financial toxicity as outlined above.

First, we conducted a univariate analysis. Ordinally scaled variables that were significantly associated with the degree of subjective financial distress per Spearman’s correlation included the net household income, global health status/quality of life, degree of direct costs, and degree of loss of income (Table 2). Lower net household income as well as lower global health status/quality of life were associated with higher subjective financial distress. In contrast, higher direct costs as well as higher loss of income correlated with higher subjective financial distress. The associations for direct costs and loss of income to subjective financial distress were amplified if assessed in relation to the net household income. This means that objective financial burden was found to be more strongly correlated to subjective financial distress in cancer patients with lower net household income.

**Table 2:** Spearman correlation analysis of subjective financial distress per question 28 of the EORTC QLQ C‑30 and ordinally scaled patient characteristics

Subjective financial distress -	*n*	Spearman rho	*p*	Lower 95% CI	Upper 95% CI
Age	98	−0.138	0.175	−0.328	0.062
Number of persons living in household	98	0.024	0.812	−0.175	0.222
Education	96	0.104	0.313	−0.098	0.298
Net household income	93	−0.221	*0.033*	−0.406	−0.018
Global health status/quality of life	98	−0.208	*0.040*	−0.391	−0.010
Degree of direct costs	81	0.250	*0.024*	0.034	0.444
Relative degree of direct costs	78	0.336	*0.003*	0.123	0.520
Degree of loss of income	95	0.560	*<* *0.001*	0.404	0.684
Relative degree of loss of income	90	0.573	*<* *0.001*	0.416	0.698

The relative degree of loss of income reflects the degree of loss of income divided by the net household income. The relative degree of direct costs reflects the degree of direct costs divided by the net household income. Italic numbers indicate statistically significant *p*-values < 0.05

*CI* confidence interval

Active (self-)employment was the only nominally scaled variable that was significantly associated with subjective financial distress per Fisher’s exact test if compared to retired patients (Table 3). Active (self-)employment was associated with greater subjective financial distress in this comparison, as indicated by the respective contingency table (Supplementary Information 2).

**Table 3:** Fisher’s exact test of subjective financial burden and nominally scaled patient characteristics

Subjective financial burden -	*n*	*p*
Male vs. female	98	0.916
Living alone vs. with partner	97	0.292
Public vs. private health insurance	97	0.386
Exempt from copayments (yes vs. no)	63	0.709
(Self-)employed vs. retired	86	*0.030*
(Self-)employed vs. unemployed	44	0.881
Retired vs. unemployed	64	0.358
Curative vs. palliative	98	0.060
Concomitant chemotherapy (yes vs. no)	98	0.702
Hospitalized vs. not hospitalized	97	0.167

Italic numbers indicate statistically significant *p*-values < 0.05

Second, we performed a multivariate analysis using an ordinal regression model. The dependent variable was the degree of subjective financial distress. Patient factors that were significantly associated with subjective financial distress per univariate analysis were included in the model as independent variables. In addition, we included age as independent variable, as there was a trend for lower age being associated with higher subjective financial distress (Table 2). Using a cauchit link function in our model, the assumption of proportional odds was met, as assessed by a full likelihood ratio test comparing the fit of the proportional odds model to a model with varying location parameters (X^2^(10) = 17.3, *p* = 0.139). The final model significantly predicted the dependent variable over and above the intercept-only model (X^2^(5) = 39.2, *p* < 0.001).

Net household income, loss of income, and direct costs remained significantly associated with subjective financial distress (Table 4).

**Table 4:** Ordinal regression analysis of subjective financial distress per question 28 of the EORTC QLQ C‑30 as dependent variable and patient characteristics as independent variables

Dependent variable: subjective financial distress						
Independent variables	Regression coefficient B	Wald‑Χ^2^	Odds ratio	*p*	Upper 95% CI	Lower 95% CI
Age	0.002	0.003	1.002	0.953	0.939	1.070
(Self-)employed (Yes)	2.831	3.348	16.965	0.067	0.817	352.11
Net household income	−1.266	6.688	0.282	*0.010*	0.108	0.736
Global health status/quality of life	−0.662	3.595	0.516	0.058	0.260	1.023
Degree of direct costs	1.021	5.299	2.777	*0.021*	1.164	6.627
Degree of loss of income	0.906	4.991	2.475	*0.025*	1.118	5.480

Italic numbers indicate statistically significant *p*-values < 0.05

*CI* confidence interval

### Patient knowledge and preferences on communication of financial burden

Lastly, we investigated the knowledge of patients concerning financial burden in the context of radiotherapy as well as the patient preferences on its communication.

**Fig. 2:**
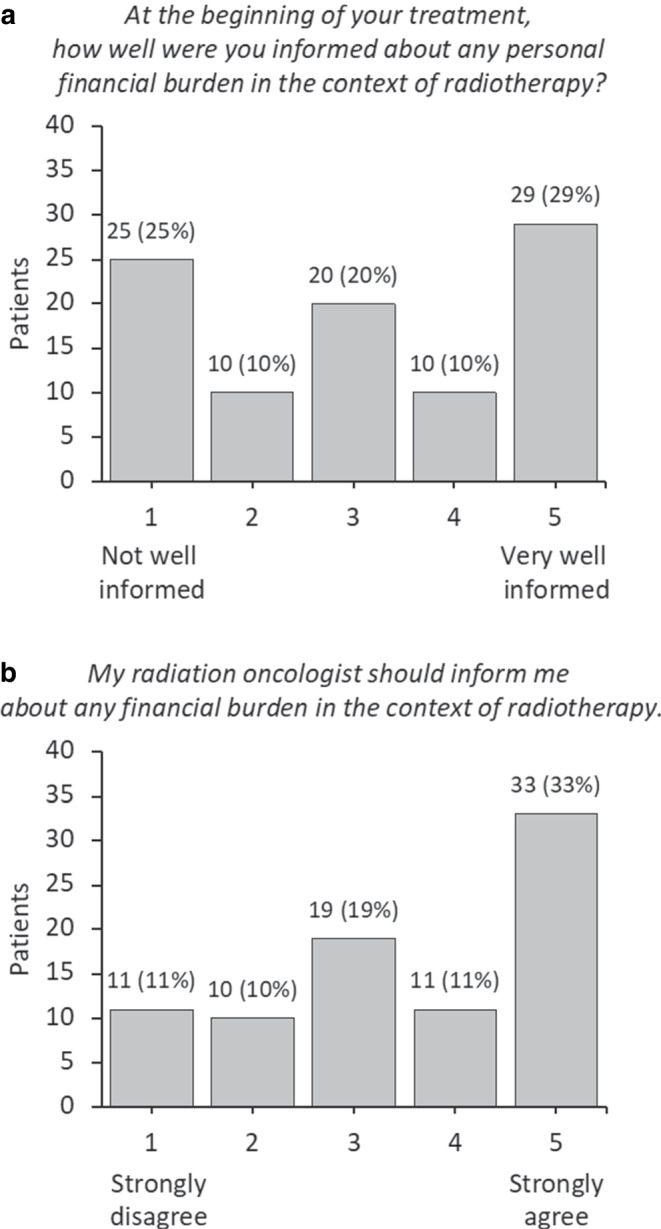
Patient knowledge of financial burden (**a**) and patient preferences on communication regarding financial burden (**b**) in the context of radiotherapy. Numbers may not add up to 100% due to missing values

Thirty-five (35%) patients felt they were not well informed, 20 (20%) patients were indifferent, and 39 (39%) patients felt well informed about any personal financial burden (Fig. 2a). Twenty-one (21%) patients disagreed, 19 (19%) patients were indifferent, and 44 (44%) patients agreed to the statement that the radiation oncologist should provide information about any financial burden (Fig. 2b).

Neither the patient knowledge of financial burden nor the patient preference on communication regarding financial burden were significantly associated with any patient characteristic or financial burden outcome per Spearman’s correlation or Fisher’s exact test.

## Discussion

This preregistered cross-sectional study provides initial evidence of financial toxicity in cancer patients treated with radiotherapy in Germany. First, in terms of objective financial burden, 68% of the patients indicated direct costs, and 25% of the patients affirmed loss of income. Subjective financial distress, the outcome closest to financial toxicity, was reported by nearly a third of the patients. Second, higher subjective financial distress was associated with lower net household income and higher objective financial burden in the multivariate analysis. Third, the patient knowledge of financial burden was unevenly distributed, whereas a relative majority welcomed information about financial burden from their radiation oncologist.

Objective financial burden has been described by a few studies in cancer patients in Germany. A cross-sectional study, for example, surveyed patients with an advanced-stage neuroendocrine neoplasm or colorectal cancer [18]. Eighty-one percent of the patients reported direct costs and 37% loss of income due to their disease. Furthermore, Büttner and colleagues followed cancer patients longitudinally that were hospitalized for treatment [19]. The patients reported mean direct costs over a period of 3 months of nearly 200 €. Although these studies assessed different patient populations, our study results appear comparable given the rates of objective financial burden mentioned above and given that most of our patients reported direct costs in the range of 101–500 €. Reasons for direct costs in our study were transportation, copayments, and nonrefundable costs for supportive care. The latter reminds us of a direct impact that we as treating radiation oncologists may have on some aspects of the financial burden of our patients.

When comparing subjective financial distress, data are again scarce for cancer patients in Germany. Furthermore, there are multiple approaches to measure and define subjective financial distress [1]. In the following, we therefore focus on studies that also used question 28 of the EORTC QLQ C‑30 questionnaire. The only comparable German study has been published by Büttner and colleagues who reported that 20% of the patients had some degree of subjective financial distress at the end of their hospital stay [19]. Perrone and colleagues summarized results from various Italian prospective trials regarding subjective financial distress [3]. At the beginning of their treatment, 18% of the cancer patients indicated a little, 6% quite a bit, and 2% very much subjective financial distress, resulting in a rate of 26% overall. A Finnish cross-sectional study surveyed cancer patients at different stages of their disease trajectory [20]. Fourteen percent of the patients reported a little, 5% quite a bit, and 2% very much subjective financial distress, resulting in a rate of 21% overall. Compared to these studies, rates in our study seem rather high, as 21% of the patients reported a little, 6% quite a bit, and 4% very much subjective financial distress, resulting in a rate of 31% overall. Therefore, financial toxicity appears also to be a prevalent issue in patients undergoing radiotherapy in Germany, and identification of risk factors is warranted.

Identification of patient factors associated with financial toxicity or subjective financial distress could aid in the early identification of patients affected by or at-risk of financial toxicity. Objective financial burden has been linked repeatedly to subjective financial distress supporting the conceptual definition of financial toxicity outlined in the introduction [1]. A Canadian longitudinal study of breast cancer patients, for example, identified loss of income as primordial factor of a perceived worsened financial situation [21]. Furthermore, the Finnish study mentioned above reported that higher direct costs correlated with increased subjective financial distress [20]. Lower household income is also a known risk factor as described, for example, by a cross-sectional study of gynecological cancer patients from Israel and the USA [22]. Therefore, our finding that direct costs, loss of income, and net household income were significantly associated with subjective financial distress upon multivariate analysis fits well into the context of the international literature. Of note, two factors showed a trend towards increased subjective financial distress: lower global health status/quality of life and active employment. In fact, lower global health status/quality of life has been associated with subjective financial distress in various studies [3, 20, 23]. The role of active employment, however, is less clear, as the Finnish study reported higher rates of subjective financial distress in unemployed persons [20]. Taken together, objective financial burden, low net household income, and possibly low global health status/quality of life appear as risk factors for subjective financial distress. Interestingly, the degree of subjective financial distress did not correlate with the patient preferences on communication of financial burden.

Concerning patient preferences, we expected a uniform agreement that information on financial burden should come from the radiation oncologist. Although a relative majority, only 44% of the patients agreed to this statement. Twenty-one percent disagreed. In addition, there was no association with any patient characteristic or financial toxicity outcome. Yet these results were reflected in a similar cross-sectional study of cancer patients in the US [16]. In this study, only 20% of the patients agreed to the statement that information on costs of care should come from their oncologist. An explanation put forward by the authors is a potential discomfort of patients to discuss costs of care with their physician. This discomfort may arise from fear that these discussions could negatively impact a patient’s treatment or the physician’s perception of the patient. Communication of costs will need to be considered and better understood to also potentially inform shared decision-making [24]. Furthermore, it will be interesting to see if patients might be more willing to discuss financial issues with social workers of the multidisciplinary care team. In this case, radiation oncologists should be aware of risk factors associated with financial toxicity, provide information as requested, and/or refer the patient to the social service if available.

Our study has limitations. The participation rate was 53.5%. We cannot rule out that patients affected by financial toxicity were more likely to participate, resulting in higher rates of financial toxicity. The rates that we have observed, however, do not seem to be gross outliers compared to the literature available so far. Furthermore, we opted for the design of a brief questionnaire to keep the patient inconvenience as low as possible. Yet additional variables might have offered a broader perspective, as psychological distress, for example, has also been associated with financial toxicity [25]. In addition, we had to rely on objective financial burden and subjective financial distress as proxy outcomes for financial toxicity. Although a multi-item questionnaire on financial toxicity with a summary effect size has been developed and a questionnaire in the setting of radiation oncology is under investigation, there are no validated German versions to date [26, 27]. The latter is crucial, as financial issues may vary per country and culture. Lastly, the question on subjective financial distress (EORTC QLQ C‑30, question 28) asked at the end of radiotherapy was intentionally not adapted in its contextual wording. Although this approach ensured the validity of the question and its comparison to other publications, we cannot rule out that factors other than radiotherapy (e.g. previous anti-cancer therapies or disease progression) may have had an influence on responses.

### Conclusions

Our study suggests that financial toxicity is so far underreported yet prevalent in cancer patients treated with radiotherapy in Germany. The awareness of risk factors associated with financial toxicity such as direct costs, loss of income, and low household income may already now help us to identify at-risk patients. Confirmatory studies are needed to firmly establish the validity of these risk factors. Furthermore, future studies should focus on the prevention of and interventions against financial toxicity aiming to improve satisfaction and outcomes of patients treated with radiotherapy.

## Supplementary Information

Supplementary Information 1 Study questionnaire

Table 2 Contingency table of the Fisher’s exact test of subjective financial burden and employment status
