# Supplementary material for: Financial toxicity in cancer patients treated with radiotherapy in Germany—a cross-sectional study
Source: Strahlenther Onkol. 2022 Apr 25;198(12):1053–61. doi: 10.1007/s00066-022-01936-z (PMC9700565; doi:10.1007/s00066-022-01936-z)
Supplement: Supplementary file 1 — Supplementary Information 1 Study questionnaire [file 66_2022_1936_MOESM1_ESM.pdf]

English translation – see below for the original version in German

Patient survey on financial burden in the context of radiotherapy

Sozio-demographic data

1.) Sex: ☐ female ☐ male

2.) How old are you? ____________ years

3.) How do you live?

☐ alone ☐ with spouse ☐ with partner

4.) How many persons are living in your household? ____ *(number, including yourself)*

5.) Your highest school graduation:

☐ None ☐ < 10 years of education *(„Hauptschulabschluss")* ☐ 10 years of education *(„Realschulabschluss")* ☐ > 10 years of education *(„Abitur")*

6.) What is your health insurance? ☐ social ☐ private

6a.) if social: Are you exempt from copayments? ☐ Yes ☐ No

Clinical data

7.) When was the cancer you are currently treated for diagnosed first?

_______/__________ *(mm/yyyy)*

8.) For which type of cancer are you currently treated with radiotherapy?

☐ Breast cancer ☐ Prostate cancer ☐ Head and Neck cancer
☐ Lung cancer ☐ Esophageal cancer ☐Rectal Cancer

☐ other: *(please note)*

9.) Did you receive chemotherapy along with your radiotherapy?

☐ Yes ☐ No *(hormone therapy excluded)*

10.) Have you been hospitalized during your radiotherapy?

☐ Yes, throughout ☐ Yes, in part ☐ No, not at all

Employment and financial data

11.) What is your current employment status?

☐ Employed ☐ Self-employed ☐ Official ☐ Unemployed ☐ Retired

12.) What is your monthly net household income? This means, the money at your disposal after deduction of taxes and social insurance contributions.

☐ < 1.300 € ☐ 1.301 – 1.700 € ☐ 1.701 – 2.600 €
☐ 2.601 – 3.600 € ☐ 3.600 – 5.000 € ☐ > 5.000 €

13.) Have you been on sick leave during your radiotherapy? ☐ Yes ☐ No

14.) Did you have any <u>loss of income</u> due to your radiotherapy?

☐ Yes ☐ No

14a.) If yes, to which degree overall?

☐ < 100 € ☐ 101 - 500€ ☐501 - 1500€ ☐ 1501 - 2500€ ☐ > 2500€

15.) Did you have any <u>additional costs</u> due to your radiotherapy? (e.g. copayments, medicines exempt from reimbursement etc.)? ☐ Yes ☐ No

15a.) If yes, to which degree overall?

☐ < 100 € ☐ 101 - 500€ ☐501 - 1500€ ☐ 1501 - 2500€ ☐ > 2500€

15b.) What were the reasons for additional costs? (*multiple choices possible*)

☐ Copayments (e.g. drugs, therapeutic products, medical aids, hospital stay etc.)
☐ Transportation
☐ Drugs/Products for supportive care to alleviate side effects of radiotherapy that are exempt from reimbursement (e.g. care lotions or washes)
☐ Alternative medicines for supportive care to alleviate side effects of radiotherapy that are exempt from reimbursement (e.g. homoeopathy)
☐ Other (e.g. PET-CT) *(please note)*:

16.) *During the past week:* has your physical condition or medical treatment caused you financial difficulties?

☐ Not at all ☐ A little ☐ Quite a bit ☐ Very much

17.) On a scale from 1 (not well informed) to 5 (very well informed):
At the beginning of your radiotherapy, how well were you informed about any personal financial burden in the context of radiotherapy?

1	2	3	4	5
Not well informed				*Very well informed*

18.) On a scale from 1 (strongly disagree) to 5 (strongly agree):
My radiation oncologist should inform me about any financial burden in the context of radiotherapy.

1	2	3	4	5
Strongly disagree				*Strongly agree*

Health status and quality of life

For the following questions please circle the number between 1 and 7 that best applies to you:

19.) How would you rate your overall <u>health</u> during the past week?

1	2	3	4	5	6	7
Very poor						*Excellent*

20.) How would you rate your overall <u>quality of life</u> during the past week?

1	2	3	4	5	6	7
Very poor						*Excellent*

Klinik für Strahlentherapie, Campus Kiel und WKK Heide
Ansprechpartner: Dr. med. Alexander Fabian
Arnold-Heller-Str. 3, 24105 Kiel
Tel.: 0431-50026501

Patientenfragebogen zur finanziellen Belastung im Rahmen der Strahlentherapie

Sozio-Demographische Aspekte

1.) Geschlecht: ☐ weiblich ☐ männlich

2.) Wie alt sind Sie? ____________Jahre

3.) Wie leben Sie?
☐ alleine ☐ mit Ehepartner/-in ☐ mit Lebenspartner/-in

4.) Wie viele Personen leben in Ihrem Haushalt? _______ *(Anzahl, Sie eingeschlossen)*

5.) Ihr höchster Schulabschluss:
☐ kein Abschluss ☐ Hauptschulabschluss ☐ Realschulabschluss ☐ Abitur

6.) Wie sind Sie krankenversichert? ☐ gesetzlich ☐ privat
6a.) falls gesetzlich: Sind Sie von Zuzahlungen befreit? ☐ Ja ☐ Nein

Angaben zur Erkrankung

7.) Wann wurde die aktuell behandelte Tumorerkrankung bei Ihnen zuerst festgestellt?
_______/__________ *(Monat/Jahr, z.B. 05/2021)*

8.) Für welche Tumorerkrankung erhalten Sie aktuell die Strahlentherapie?
☐ Brustkrebs ☐ Prostatakrebs ☐ Rachen-/Mundhöhlen- / Kehlkopfkrebs
☐ Lungenkrebs ☐ Speiseröhrenkrebs ☐ Darmkrebs

☐ sonstige, nicht aufgeführte Tumorerkrankung:
(bitte notieren)

9.) Haben Sie parallel zur aktuellen Strahlentherapie eine Chemotherapie erhalten?
☐ Ja ☐ Nein *(Hormontherapie zählt nicht dazu)*

10.) Erfolgte die Strahlentherapie stationär, d.h. waren Sie stationär im Krankenhaus aufgenommen? ☐ Ja, durchgehend ☐ Ja, teilweise ☐ Nein, gar nicht

Berufliche und finanzielle Aspekte

11.) Wie ist Ihr aktueller Erwerbsstatus?
☐ Angestellt ☐ Selbstständig ☐ Beamter/in ☐ nicht erwerbstätig ☐ berentet

12.) Wie hoch ist ihr gesamtes monatlich verfügbares Haushalts-Nettoeinkommen, also das Geld, das nach Abzug der Steuern und Sozialversicherungsbeiträge dem gesamten Haushalt übrigbleibt?
☐ unter 1.300 € ☐ 1.301 – 1.700 € ☐ 1.701 – 2.600 €
☐ 2.601 – 3.600 € ☐ 3.600 – 5.000 € ☐ über 5.000 €

13.) Waren/sind Sie während der Strahlentherapie krankgeschrieben? ☐ Ja ☐ Nein

Bitte wenden **Rückseite ➔**

14.) Sind Ihnen in Folge Ihrer aktuellen Behandlung in der Strahlentherapie Einkommenseinbußen entstanden? ☐ Ja ☐ Nein

14a.) Wenn ja, in welcher Höhe insgesamt?

☐ unter 100 € ☐ 101 - 500€ ☐ 501 - 1500€ ☐ 1501 - 2500€ ☐ über 2500€

15.) Haben Sie in Folge Ihrer aktuellen Behandlung in der Strahlentherapie höhere Ausgaben (z.B. Zuzahlungen, nicht-erstattete Arzneien etc.)? ☐ Ja ☐ Nein

15a.) Wenn ja, in welcher Höhe insgesamt?

☐ unter 100 € ☐ 101 - 500€ ☐ 501 - 1500€ ☐ 1501 - 2500€ ☐ über 2500€

15b.) Wodurch fallen Ihnen höhere Ausgaben an? (*Mehrfachnennungen möglich*)

☐ Zuzahlung (z.B. Arznei- / Heil- / Hilfsmittel, Krankenhausaufenthalt etc.)
☐ Fahrtkosten
☐ Arzneimittel/Pflegeprodukte zur Linderung von Nebenwirkungen der Strahlentherapie, die nicht erstattet werden (z.B. pflegende Salben/Spülungen)
☐ Alternativmedizinische Mittel zur Linderung von Nebenwirkungen der Strahlentherapie, die nicht erstattet werden (z.B. Homöopathie)
☐ Sonstiges (z.B. PET-CT) *(bitte notieren)*:

16.) *Während der letzten Woche:* Hat Ihr körperlicher Zustand oder Ihre medizinische Behandlung für Sie finanzielle Schwierigkeiten mit sich gebracht?

☐ überhaupt nicht ☐ wenig ☐ mäßig ☐ sehr

17.) Auf einer Skala von 1 (nicht gut informiert) bis 5 (sehr gut informiert):
Wie gut waren Sie bei Behandlungsbeginn über etwaige persönliche finanzielle Belastungen im Rahmen der Strahlentherapie informiert?

1 2 3 4 5

nicht gut informiert *sehr gut informiert*

18.) Auf einer Skala von 1 (stimme gar nicht zu) bis 5 (stimme voll zu):
Mein(e) Strahlentherapeut(in) sollte mich über etwaige persönliche finanzielle Belastungen im Rahmen der Strahlentherapie informieren.

1 2 3 4 5

stimme gar nicht zu *stimme voll zu*

Gesundheit und Lebensqualität

Bitte kreuzen Sie bei beiden folgenden Fragen die Zahl zwischen 1 und 7 an, die am besten auf Sie zutrifft:

19.) Wie würden Sie insgesamt Ihren Gesundheitszustand während der letzten Woche einschätzen?

1 2 3 4 5 6 7

sehr schlecht *ausgezeichnet*

20.) Wie würden Sie insgesamt Ihre Lebensqualität während der letzten Woche einschätzen?

1 2 3 4 5 6 7

sehr schlecht *ausgezeichnet*

Vielen Dank für Ihre Zeit und Teilnahme

Bitte geben Sie den Fragebogen im verschlossenen Umschlag beim Klinikpersonal ab.
