# Supplementary material for: Financial toxicity in cancer patients treated with radiotherapy in Germany—a cross-sectional study
Source: Strahlenther Onkol. 2022 Apr 25;198(12):1053–61. doi: 10.1007/s00066-022-01936-z (PMC9700565; doi:10.1007/s00066-022-01936-z)
Supplement: Supplementary file 2 — Table 2 Contingency table of the Fisher’s exact test of subjective financial burden and employment status [file 66_2022_1936_MOESM2_ESM.docx]

| **Supplementary information 2** Contingency table of the Fisher’s exact test of subjective financial burden and employment status | | | | | | | | | |
| --- | --- | --- | --- | --- | --- | --- | --- | --- | --- |
|  | | | | **(Self-)employed vs. retired** | | | |  | |
| **Subjective financial burden** | |  | | **(Self-)employed** | | **Retired** | | **Total** | |
| Not at all |  | Count |  | 19.000 |  | 41.000 |  | 60.000 |  |
|  |  | Expected count |  | 23.023 |  | 36.977 |  | 60.000 |  |
|  |  | % within row |  | 31.667 % |  | 68.333 % |  | 100 % |  |
|  |  | % within column |  | 57.576 % |  | 77.358 % |  | 69.767 % |  |
| A little |  | Count |  | 7.000 |  | 10.000 |  | 17.000 |  |
|  |  | Expected count |  | 6.523 |  | 10.477 |  | 17.000 |  |
|  |  | % within row |  | 41.176 % |  | 58.824 % |  | 100 % |  |
|  |  | % within column |  | 21.212 % |  | 18.868 % |  | 19.767 % |  |
| Quite a bit |  | Count |  | 3.000 |  | 2.000 |  | 5.000 |  |
|  |  | Expected count |  | 1.919 |  | 3.081 |  | 5.000 |  |
|  |  | % within row |  | 60.000 % |  | 40.000 % |  | 100% |  |
|  |  | % within column |  | 9.091 % |  | 3.774 % |  | 5.814 % |  |
| Very much |  | Count |  | 4.000 |  | 0.000 |  | 4.000 |  |
|  |  | Expected count |  | 1.535 |  | 2.465 |  | 4.000 |  |
|  |  | % within row |  | 100 % |  | 0 % |  | 100 % |  |
|  |  | % within column |  | 12.121 % |  | 0 % |  | 4.651 % |  |
| Total |  | Count |  | 33.000 |  | 53.000 |  | 86.000 |  |
|  |  | Expected count |  | 33.000 |  | 53.000 |  | 86.000 |  |
|  |  | % within row |  | 38.372 % |  | 61.628 % |  | 100 % |  |
|  |  | % within column |  | 100 % |  | 100 % |  | 100 % |  |
|  | | | | | | | | | |
